# Serum as a surrogate for salivary IgG: comparison of antigen-specific serum and salivary IgG in sheep

**DOI:** 10.3389/fimmu.2026.1797719

**Published:** 2026-05-20

**Authors:** Juliana Yeung, Rina Hannaford, Tania Wilson, Tim Ferguson, Sofia Khanum, Tom N. McNeilly, Peter H. Janssen, D. Neil Wedlock

**Affiliations:** 1 Lucidome Bio, Palmerston North, New Zealand; 2 Bioeconomy Science Institute – AgResearch Group, Palmerston North, New Zealand; 3 Disease Control Department, Moredun Research Institute, Penicuik, United Kingdom

**Keywords:** correlation, ELISA, IgG, IgG1, IgG2, saliva, serum, sheep

## Abstract

**Background:**

IgG is a major antibody isotype in serum and is transferred to saliva in humans and ruminants. Vaccination strategies against rumen-dwelling organisms such as methanogens aim to induce high levels of antibody in saliva, yet saliva is difficult to collect in sufficient volumes for routine analysis. This study evaluated whether serum could serve as a surrogate for saliva in assessing antigen-specific IgG responses in sheep.

**Methods:**

Paired serum and saliva samples were collected from animals vaccinated with antigens of varying complexity: a single recombinant protein (SP1), two protein cocktails, and a mixture of five methanogen strains (Mix5). Forty-one paired serum and saliva samples were analyzed. Antigen-specific IgG, IgG1, and IgG2 titers in serum and saliva were compared using ELISA and western blotting. Antibody avidity was determined using a chaotropic agent in the ELISA.

**Results:**

Serum and saliva showed strong overall correlation for antibody titers, and the specificities of antibody-antigen interaction were very similar for serum and salivary antigen-specific IgG, IgG1 and IgG2 antibodies. For antibody avidity, the data supported a broad positive relationship between serum and salivary IgG; however, additional methodological refinement and validation are required before this relationship can be confidently extrapolated across antigen types.

**Conclusions:**

Collectively, these results demonstrate that serum provides a robust and practical surrogate for assessing key immunological properties of antigen-specific IgG in sheep saliva.

## 1 Introduction

Antibodies are a crucial component of the humoral adaptive immune response, with a role in neutralization, complement fixation, and opsonization of disease-causing molecules, viruses and cells (1). IgG is the dominant antibody isotype in serum and is present in saliva of humans (2, 3), and ruminants (4–7). In humans, IgG (and monomeric IgA of similar molecular size) enters the oral cavity from the peripheral blood circulation by passive diffusion through gingival crevices, while the majority of salivary IgA is produced by plasma cells present in the salivary gland (3). In sheep, IgG1 and IgG2 detected in saliva is derived from plasma (8), in contrast to IgA and IgM, which are produced locally at mucosal sites, and transported into respiratory tract secretions and saliva (9).

Sheep and cattle possess two IgG subclasses (IgG1 and IgG2), with cattle expressing a third subclass, IgG3 (4, 10, 11). In sheep, IgG3 has historically been defined on the basis of γ−chain sequence and serology as an IgG allotype rather than as a functionally distinct IgG subclass equivalent to IgG1 or IgG2 (4, 12). Ovine and bovine IgG1 and IgG2 have similar roles, and the main difference with each subtype is their interactions with immune cells and receptors when neutralizing pathogens and facilitating phagocytosis. IgG1 plays a significant role in passive immunity through colostrum and milk, binding to specific Fc receptors on immune cells to neutralize viruses and toxins (10, 11, 13). IgG2 is effective for opsonization and promoting phagocytosis, particularly by neutrophils, and is important for promoting antibody-dependent cellular cytotoxicity (ADCC) (10, 13–15). Unique to IgG2 is its specific ability to bind to Fcγ2R receptors on immune cells, indicating a specialized role in immune responses compared to IgG1 (12). At present, limited availability of validated ovine IgG3−specific reagents precludes robust analysis of IgG3 responses. For this reason, the current study focuses on total ovine IgG, IgG1, and IgG2 responses in both serum and saliva.

Blood is commonly used in serological assays that evaluate systemic immune responses. Collecting small volumes of saliva is relatively less invasive than obtaining blood samples but few molecules can be effectively analyzed in saliva (16, 17) because the analytes of interest are only present at low concentrations. Saliva has been considered as a medium to determine successful transfer of colostrum IgG to calf serum (17), but the amount of IgG detectable in saliva of calves (17) and adult sheep and cattle (6, 7) is below sensitivity limits of many sero-diagnostic assays. Collecting sufficient saliva for studying the interaction of antibodies with antigens on a routine basis is impractical, due to increased animal handling and the need for specialized equipment (17, 18). In contrast, only a small amount of serum is required for serological assays, and it is widely used in veterinary diagnostics to evaluate IgG-antigen interactions (17, 19), based on the assumption that serum IgG has similar immunological properties to salivary IgG. This study aimed to directly test this assumption by comparing the magnitude and properties of antigen−specific IgG responses in paired serum and saliva samples from vaccinated sheep.

Vaccination of farmed ruminants has been proposed as a mechanism to modulate the activities of rumen-dwelling microbes. Such approaches have been proposed for limiting the impacts of lactate-forming bacteria during feed transitions to high-grain diets (20), for targeting methanogens to reduce their methane emissions (21, 22), lowering protozoal populations to increase wool production (23), and to decrease the activity of ruminal ureases to decrease nitrogen release kinetics (24). Vaccinating sheep and cattle should generate antigen-specific antibodies in their serum and saliva, predominately IgG. The salivary antibodies would enter the rumen where they would interact with the target microbes or enzymes to lower their activity. In this context, salivary IgG represents the biologically relevant antibody population for binding-mediated interactions with rumen microbes. Ruminants produce large volumes of saliva, and it is the main vehicle for transporting antigen-specific IgG into the rumen. For example, cattle produce 1.5-2.5 rumen volumes of saliva each day (25) and vaccinated animals would continuously deliver antigen-specific IgG into the rumen. Experimental vaccination studies have shown that this continuous salivary flow delivers large numbers of antigen−specific IgG molecules to the rumen; estimated to exceed 10^4^ molecules per target microbe and that a substantial fraction of these antibodies retain antigen−binding activity for several hours under rumen−like conditions (6, 7).

In this study, we compared antigen-specific IgG responses and their subclasses (IgG1 and IgG2) in paired saliva and serum samples from sheep vaccinated with antigens of differing complexity: a single recombinant protein (SP1), two different mixtures of recombinant proteins (Cocktail 1 and Cocktail 2), and a mix of five cultured methanogens (Mix5). Our primary objective was to assess whether serum IgG titers and antigen specificity reliably reflect those observed in saliva. In addition, we explored the relationship between serum and salivary IgG avidity as a secondary measure of antibody–antigen interaction. Since avidity measurements may be influenced by assay characteristics and limited dynamic range, these analyses were interpreted cautiously. Serum cannot be considered biologically equivalent to saliva; however, it can serve as a robust indicator of antigen−specific salivary IgG responses during vaccine optimization.

## 2 Materials and methods

### 2.1 Animals

Female Romney cross sheep aged 6–12 months were sourced from a commercial farm in the North Island of New Zealand. All animals were grazed on pastures at the same farm (Aorangi Farm, AgResearch, New Zealand) with water ad libitum and monitored weekly for normal appearance and behavior. All animal procedures were approved by the AgResearch Grassland’s Animal Ethics Committee (New Zealand) prior to commencement of the study. Study identifiers and details of the trials are listed in **Supplementary Table 1**.

### 2.2 Preparation of recombinant proteins

Recombinant proteins were produced by GenScript (Piscataway, New Jersey, USA). Briefly, proteins were expressed in *Escherichia coli* BL21 with a His-tag. Protein expression in cultures was induced with isopropyl β-D-1-thiogalactopyranoside (IPTG), and the recombinant proteins were purified using Fast Protein Liquid Chromatography (FPLC). Proteins were analyzed by SDS-PAGE and western blotting with a mouse-anti-His monoclonal antibody to confirm correct molecular weight and purity. Protein concentration was determined using a Bradford protein assay according to the manufacturer’s instructions (Thermo Fisher Scientific, Auckland, New Zealand). The identity of the recombinant proteins is proprietary to Lucidome Bio, but the single protein and two cocktails did not share any proteins in common.

### 2.3 Growth of methanogens and preparation of total cell lysate

Five different isolates of *Methanobrevibacter* species from Lucidome Bio’s culture collection, which included *Methanobrevibacter ruminantium* M1 (DSM 1093), were grown in BY medium as described previously (26, 27). Individual cultures were grown to late-logarithmic or early stationary phase and harvested by centrifugation. The cells were washed in PBS (137 mM NaCl, 2.7 mM KCl, 8.1 mM; Na_2_HPO_4_, 1.8 mM KH_2_PO_4_, pH 7.3 with HCl).

A total cell lysate was prepared from 100 mg wet weight of *M. ruminantium* M1 cell pellet by grinding the cell pellet with a mortar and pestle in liquid nitrogen. Following addition of 500 µL of 4 mM Tris-HCl (pH 8) containing 2× cOmplete™ EDTA-free protease inhibitor cocktail (Sigma-Aldrich, St. Louis, Missouri, USA), the cells were sonicated on ice for a total of 3 min at 30 s intervals using a microtaper tip at 40% intensity (VCX 750 Ultrasonic Processor; Sonics, Newtown, Connecticut, USA) with 1 min rests on ice in between. The lysate was centrifuged at 3,000 × *g* for 10 minutes (Centrifuge 5417C; Eppendorf, Hamburg, Germany) and the supernatant (total cell lysate) was stored at −20 °C prior to use.

### 2.4 Vaccination of sheep, sampling and preparation of serum and saliva samples

Paired serum and saliva samples were obtained from a total of 41 sheep vaccinated with the different antigens in three separate vaccination trials. In trial 1, a group of sheep (n = 14) were vaccinated with a mixture of different recombinant proteins (Cocktail 1). In trial 2, a group of sheep (n = 12) were administered a different mixture of different recombinant proteins (Cocktail 2). In trial 3, groups of sheep were vaccinated with a single recombinant protein (SP1; n = 6 sheep), a mixture of the five strains of methanogens (Mix5; n = 9) or given adjuvant alone as controls (n = 3). Methanogens were inactivated with hydrogen peroxide prior to use as vaccines and the inactivation method was validated in-house and approved by AgResearch Grassland’s Animal Ethics Committee (New Zealand). Each dose of vaccine consisted of one of the following: Cocktail 1 or Cocktail 2 of recombinant proteins, protein SP1, approximately 10^9^ cells of each of the five strains in a mix, or adjuvant control alone. These antigens were formulated with Montanide ISA61 (SEPPIC, Paris, France). The composition of the vaccines used in this study are proprietary to Lucidome Bio. Vaccines were administered intramuscularly as a 2-mL dose in the anterior region of the neck. Animals were revaccinated with the same vaccines after 4 weeks.

At 2 or 3 weeks after the second vaccination, paired blood and saliva samples were collected from each animal. Blood samples were obtained by jugular venepuncture into serum separation tubes (Vacutainer SST™ II Advance, 8.5 mL, gold-top; BD Biosciences, Franklin Lakes, New Jersey, USA). Samples were allowed to clot and centrifuged at 3,500 rpm for 10–15 min, after which serum was transferred into labelled microcentrifuge tubes and stored at −20 °C until use. Repeated freeze–thaw cycles were avoided, with samples thawed only once where possible. Small quantities of saliva were collected from all animals using a cotton swab placed in the mouth. The swab was placed in a 5-mL Salivette saliva collection tube (Sarstedt, Nümbrecht, Germany), centrifuged at 2,000 × *g* for 10 min and the flow-through collected. No visual blood contamination was observed in any of the saliva samples.

### 2.5 Antigen-specific ELISA assays

Antigen−specific antibody responses in serum and saliva were measured using two closely related ELISA formats. A single standardized indirect ELISA was applied across all antigens to quantify total IgG, ensuring consistent within−antigen comparisons of paired serum and saliva. Subclass responses (IgG1, IgG2) were measured using a similar, purpose−optimized, collaborator-validated subclass ELISA (NcNeilly Laboratory, Moredun Research Institute). Neither assay was independently optimized for individual antigens. This preserves comparability within each antigen but limits direct quantitative comparison of absolute responses between antigens of differing composition or complexity.

The method used was described by Subharat et al. (6) with minor modifications. Briefly, high binding Nunc ELISA plates (Thermo Fisher Scientific) were coated overnight with 50 µL per well of antigen (2 µg SP1/mL, 10 μg whole cell methanogen mix/mL, or 1 µg of each protein combined as a mixture for Cocktails 1 or 2 per mL in 50 mM carbonate-bicarbonate buffer, pH 9.6 (Sigma-Aldrich) at 4 °C. The plates were blocked with PBS containing 1% (w/v) casein for 1 h at room temperature (RT).

Serum and saliva were collected for each sheep at week 0, just before vaccination, and assessed at the lowest dilution, 1:200 (serum) and 1:2 (saliva), for background immune responses. Two-fold serial dilutions of serum and saliva, starting at 1:6,400 (serum) and 1:8 (saliva) in PBS were added to duplicate wells and incubated for 1 h at RT. The plates were washed with PBS containing 0.1% Tween 20 (PBST). Then, 50 μL of horse radish peroxidase (HRP)-conjugated donkey anti-sheep IgG (Jackson ImmunoResearch, West Grove, Pennsylvania, USA; 1:6,000 dilution) in PBS containing 1% (w/v) casein was added to each well. The plates were incubated for 30 min at 37 °C, washed with PBST, and 50 μL of 3,3′,5,5′-tetramethylbenzidine (TMB) substrate (BD Biosciences) was added per well before the plates were incubated in the dark for 20 min. The reactions were stopped by addition of 25 μL of 0.5 M H_2_SO_4_ per well, and the absorbance at 450 nm was read with a VERSAmax microplate reader (Molecular Devices, San Jose, California, USA).

Subclass−specific antibody responses were measured in parallel using a related ELISA protocol with defined modifications to reagents and blocking conditions. The secondary mouse monoclonal antibodies, anti-ovine IgG1 (clone McM1) and anti-ovine IgG2 (clone McM2), were produced by ImmunoTools GmbH (Friesoythe, Germany). The coating buffer was 0.5 M sodium carbonate buffer, pH 9.6 and the blocking buffer was 3% (w/v) fish gelatine (Sigma-Aldrich) in PBS. Anti-ovine IgG1 (1:1,000) or anti-ovine IgG2 (1:2,000) in 3% fish gelatine in PBS were added to the wells, then plates were incubated for 1 h at 37 °C. A tertiary antibody was used to measure binding. This was rabbit polyclonal anti-mouse pan IgG-HRP (Agilent DAKO, Santa Clara, California, USA), diluted 1:1,000 in PBST, added at 50 µL per well, and incubated for 1 h at 37 ˚C. Color reactions were developed using Sigma-Fast o-phenylenediamine dihydrochloride (OPD) substrate (Sigma-Aldrich) and stopped using by addition of 25 µL of 2.5 M H_2_SO_4_ per well.

Control samples were included in each ELISA plate. The positive control sample was made with pooled sera from sheep vaccinated with an in-house antigen and plated at a known concentration (1:800 dilution) that produced a consistent positive result. The negative control wells were treated the same as the other wells, but without addition of antigens and had PBS added in place of serum to provide the background absorbance of the assay.

### 2.6 Determination of antibody avidity

Antibody avidity was measured by ELISA using sodium thiocyanate (NaSCN) as a chaotropic agent to disrupt antibody-antigen interactions. An ELISA assay similar to the one described above was used to measure antigen-specific IgG. The chaotropic agent (4 M NaSCN prepared in PBS) or just PBS (control) was added after the addition of saliva or serum. After 30 min incubation at 18 °C, the plates were washed three times with PBST. Horse radish peroxidase (HRP)-conjugated donkey anti-sheep IgG (Jackson ImmunoResearch; 1:6,000 dilution), in PBS containing 1% (w/v) casein (50 µL) was added to each well and the plates were incubated for 30 min at 37 °C. As described above, the plates were washed with PBST, followed by addition and incubation with 50 μL per well of TMB substrate and the reactions were stopped by addition of 25 µL of 0.5 M H_2_SO_4_ per well. The absorbance at 450 nm was read with a VERSAmax microplate reader (Molecular Devices). The avidity index (AI) was calculated based on the area under the antibody titration curve (AUC) as described in Section 2.8.

Antibody avidity was quantified using a chaotropic ELISA approach, with avidity index (AI) calculated as the ratio of the area under the fitted antibody titration curve (AUC) in the presence versus absence of sodium thiocyanate. Because AI values are derived from fitted curves across a limited dilution range, estimates may be sensitive to curve−fit uncertainty and constrained dynamic range, particularly for saliva samples at low or high signal intensities. Accordingly, avidity results were interpreted cautiously and considered exploratory. Curve fitting was used to reduce experimental noise within each sample type rather than to establish quantitative comparability between serum and saliva, and avidity measurements were therefore treated as a secondary, non−determinative component of the analysis.

### 2.7 SDS-PAGE and western blotting

Samples (0.5 μg) of SP1 or (6 μg) of total cell lysate were prepared in NuPAGE LDS sample buffer (Thermo Fisher Scientific) containing 50 mM dithiothreitol (Thermo Fisher Scientific) and incubated at 37 °C for 40 min prior to loading onto a 4-12% gradient Bis-Tris gel (Thermo Fisher Scientific). SeeBlue 2 plus (Thermo Fisher Scientific) was the molecular weight marker used for all SDS-PAGE gels and western blots. Electrophoresis was performed in NuPAGE SDS MOPS running buffer (Thermo Fisher Scientific) at 160 V. The separated proteins were transferred to PVDF membranes (Thermo Fisher Scientific) and blocked overnight at 4 °C in 20 mM Tris, 150 mM NaCl, pH 7.6 with HCl, 0.2% (v/v) Tween 20 (TBS-0.2T) with 7% (w/v) skim milk (Pams, Auckland, New Zealand). The membranes were incubated at room temperature with antigen-specific sera or saliva or pooled control sera diluted in TBS-0.2T containing 5% (w/v) skim milk. A 1/16 dilution of saliva was used for both SP1 and Mix5, and a 1/16,000 and 1/32,000 dilution of serum was used for SP1 and Mix5, respectively. Each blot was washed with TBS-0.5T (same as TBS-0.2T but with 0.5% (v/v) Tween 20) and then incubated with rabbit HRP-conjugated anti-sheep antibody (Jackson ImmunoResearch; 1:5,000 dilution) in TBS-0.2T. The blots were treated with Clarity ECL (Bio-Rad, Carlsbad, California, USA) following the manufacturer’s directions and images captured on a ChemiDoc system (Bio-Rad). Western blots performed on sera and saliva samples from each group of animals were exposed to chemiluminescence at the same time and the images were captured in one exposure. This enabled qualitative direct comparisons between matched serum and saliva from each animal. The purity and integrity of recombinant antigens were confirmed by SDS−PAGE prior to immunological analyses. Representative SDS−PAGE gels of recombinant SP1 (**Supplementary Figure 1A**) and total cell lysate (**Supplementary Figure 1B)** are shown in **Supplementary Materials**.

### 2.8 Statistical analyses

All statistical analyses were carried out using R software (version 4.2.1; http://www.R-project.org, R Foundation for Statistical Computing, Vienna, Austria).

#### 2.8.1 Regression

We started by fitting four-parametric logistic (4PL) regression models to the raw ELISA titration curves using the drc package in R (28). A 4PL model is defined as


$$y(x;b,l,u,e)=\;l+\;\frac{u-l}{1+\exp(b[\log(x)-\log(e)])}$$

where *y* and *x* are the response (absorbance at 450 nm) and dilution levels, respectively.

Typically, the response is a decreasing function of the dilution level. In this case, *u* is the maximum value that can be obtained, *l* is the minimum value that can be obtained, and *e* is the point of inflection, that is, the point on the curve halfway between the minimum and maximum value. This parameter is referred to as the “ED50” parameter. The final parameter is the slope *b* that describes the steepness of the curve at the ED50 point.

When comparing the serum with the saliva samples, we used the ED50 parameter as a biologically meaningful measure of the strength of the immune response. As described by Keshtkar et al. (29), the ED50 value is derived as


$$ED50=\;{\left(\frac{e^{b}u}{u-2l}\right)}^{\frac{1}{b}}$$

According to this equation, equality only holds if the minimum, *l*, is zero. Given that in our samples the parameter estimates for the lower limit were non-zero, we would produce slightly different ED50 values that take the lower and upper limit into account. We referred to these adjusted values as absolute ED50 values, aED50.

#### 2.8.2 Correlation analysis

Correlation analyses were performed to assess the relationship between paired serum and salivary antibody responses. For each vaccine group, two sets of absolute ED50 (aED50) values were calculated from ELISA titration curves, one for serum and one for saliva samples. Pearson’s product–moment correlation coefficient was used to evaluate linear associations between serum and salivary measurements, which is appropriate when approximate linearity and the absence of extreme outliers can be assumed.

For each set of measurements, the Shapiro–Wilk Normality Test was used to assess the distribution of aED50 values, and scatter plots were examined to visually confirm linear covariation and identify any pronounced outliers. Correlation analyses were performed both across pooled datasets and within individual vaccine groups to evaluate the consistency of serum–saliva relationships across antigens of differing composition and complexity. Interpretation focused primarily on within−vaccine−group analyses to account for biological and quantitative differences between antigens. Correlation analyses were used to assess the strength and consistency of association between paired serum and salivary measurements; however, correlation does not constitute evidence of quantitative agreement or measurement equivalence.

#### 2.8.3 Avidity

ELISA titration curves were modelled using the 4PL regression model to assess the avidity of IgG antigen-specific antibodies in sera and saliva. The fitted data within the corresponding data range as the raw data collected from titration curves were used for calculations. The avidity index (AI) was calculated based on the area under the antibody titration curve (AUC) method described by Perciani et al. (30) with the modification that fitted data were used instead of raw data. The AI calculated by the AUC method using fitted data was viewed as more accurate in our study since it minimized data noise observed from titration curves. Correlation analysis (data not shown) was completed to verify that the fitted data accurately represented the observations from raw data. AI values were calculated from the ratio of areas under the fitted curves (absorbance at 450 nm versus log2 of antibody dilution with and without addition of chaotropic agent). The formula used to calculate AI according to Perciani et al. (30) is AI = AUC_treated/AUC_control. To compare the avidity of serum IgG versus saliva IgG, the avidity indices were analyzed using Pearson’s correlation as described above.

## 3 Results

### 3.1 Comparison of antigen-specific IgG responses in saliva and serum

**Figure 1:**
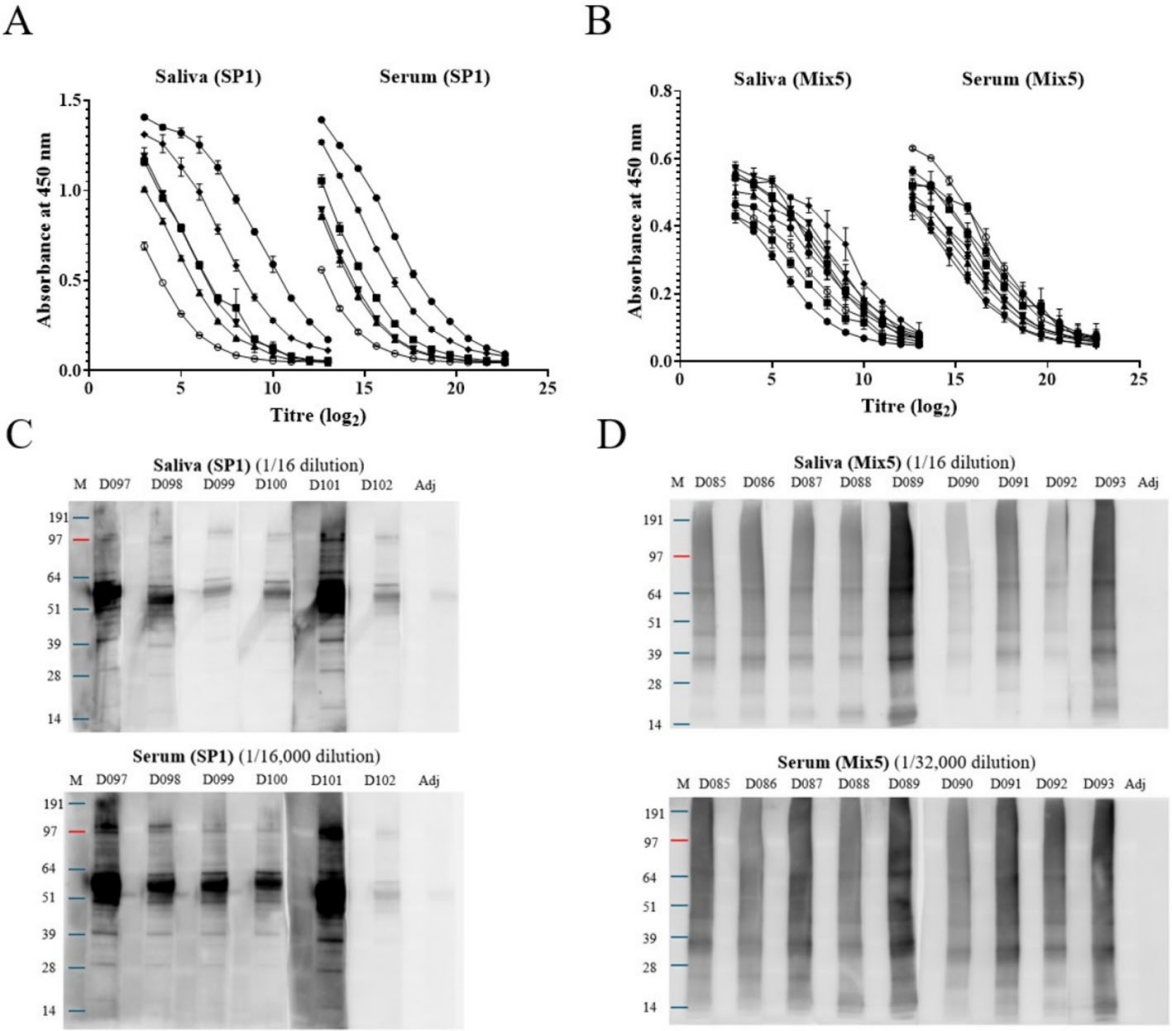
Comparison of IgG antibody responses in saliva and serum. ELISA titration curves of antigen-specific IgG antibodies in saliva and sera from sheep vaccinated with a single recombinant protein, SP1 **(A)**, or a mix of whole cell antigens from five methanogens, Mix5 **(B)**. Mean (± SE) of duplicate determinations are presented. The same symbol is used for individual animals in the titration curves with saliva and serum within each panel. Western blots **(C)** were performed with serum (1:16,000 dilution) and saliva (1:16 dilution) from sheep vaccinated with SP1, against recombinant protein SP1. 0.5 µg of recombinant SP1 was loaded onto each lane. D097 to D102 refer to individual sheep. Western blots **(D)** were performed with serum (1:32,000 dilution) and saliva samples (1:16 dilution), from sheep vaccinated with Mix5, against antigens in a total cell lysate from *M. ruminantium* M1, one of the five methanogens in the Mix5 vaccine mixture. Total cell lysate protein (6 µg) was loaded to each lane. D085 to D093 refer to individual sheep in **(D)**. Molecular weight marker was noted as M, and adjuvant controls was abbreviated as Adj, on the western blots presented.

Antigen-specific salivary and serum IgG antibodies from animals vaccinated with a protein (SP1) and a mix of methanogen isolates (Mix5) showed broadly similar titration curves when measured by ELISA (**Figures 1A, B**). Absolute ED50 values (aED50) were calculated from the ELISA titration curves (log_2_) from the same groups. Differences between aED50 serum (log_2_) values and aED50 saliva (log_2_) values are summarized in **Table 1**. These values were used to calculate the ratio of specific antibodies in serum to antibodies in saliva. On average, antigen−specific IgG levels were substantially higher in serum than in saliva, with an average ~500−fold difference in aED50 values. Animals that produced a weaker IgG response in serum also had consistently lower antigen-specific IgG in saliva. Conversely, animals with higher antibody responses in serum had consistently higher levels of antigen-specific IgG in saliva. This was also found with sera and saliva from animals vaccinated with Cocktails 1 and 2 (**Supplementary Figure 2**). A similar trend was observed for the IgG1 and IgG2 subtypes (**Table 1**, **Supplementary Figures 3**, **4**). As with total IgG, antigen−specific IgG1 and IgG2 levels were substantially higher in serum than saliva, with mean differences of approximately 600−fold and 1000−fold, respectively. There were no major differences in the relative IgG, IgG1, and IgG2 responses across vaccine groups; the serum-to-saliva aED50 ratios remained consistent, indicating a stable relative relationship between the detectable immune response in serum and saliva (**Table 1**). All aED_50_ values for each serum and saliva pairing evaluated for IgG, IgG1 and IgG2 response can be found in the Supplementary material (**Supplementary Tables 2**-**4**).

**Table 1:** Differences between aED50 serum (log_2_) values and aED50 saliva (log_2_) values for IgG, IgG1, and IgG2 assessed for each vaccine group.

Groups	IgG			IgG1			IgG2		
	Mean	Max	Min	Mean	Max	Min	Mean	Max	Min
Cocktail 1	9.90	11.51	9.17	9.81	11.91	8.18	9.73	11.82	8.39
Cocktail 2	8.57	9.68	6.42	8.81	10.16	6.78	12.05	13.44	9.88
SP1	8.43	9.08	7.46	9.21	10.23	8.06	9.04	9.67	8.71
Mix5	8.61	9.82	7.22	9.05	9.97	8.03	8.83	9.52	7.73
All groups	8.99	11.51	6.42	9.27	11.91	6.78	10.06	13.44	7.73

Western blotting indicated that antibodies in paired serum and saliva from individual animals have similar patterns of immuno-reactivity. Antibodies in saliva (tested at 1:16 dilution) and serum (tested at 1:16,000 dilution) produced against SP1 specifically detected the recombinant protein with comparable antigen recognition despite differing sample dilutions (**Figure 1C**). Likewise, antibodies in saliva (tested at 1:16 dilution) and serum (tested at 1:32,000 dilution) produced against the Mix5 methanogens showed a similar reactivity against the antigens in a total cell lysate prepared from cells *M. ruminantium* M1, one of five methanogens in the Mix5 vaccine (**Figure 1D**). The western blots for each group (SP1, Mix5, or adjuvant control), using the serum and saliva pairs, were exposed to chemiluminescence at the same time and the image for each group was captured in one exposure to permit a direct comparison between the serum and saliva responses. Animals with strong antibody responses showed consistently higher signal in both serum and saliva Western blots, while weak responders exhibited lower signal, with comparable banding patterns across both sample types. As expected, no immune response observed in western blots using serum or saliva samples from the adjuvant control animals. This pattern was also observed with the ELISA titration curves (**Figures 1A, B**), where the strongest and weakest responders were consistent between the serum and saliva pairs. Overall, the consistent rank ordering and matching antigen−recognition patterns across assays show that serum and saliva behave similarly in these foundational measures, allowing more complex relationships to be examined in the following sections.

### 3.2 Correlation analysis of aED50 data for antigen-specific IgG responses in serum describes antigen-specific IgG responses in saliva

**Figure 2:**
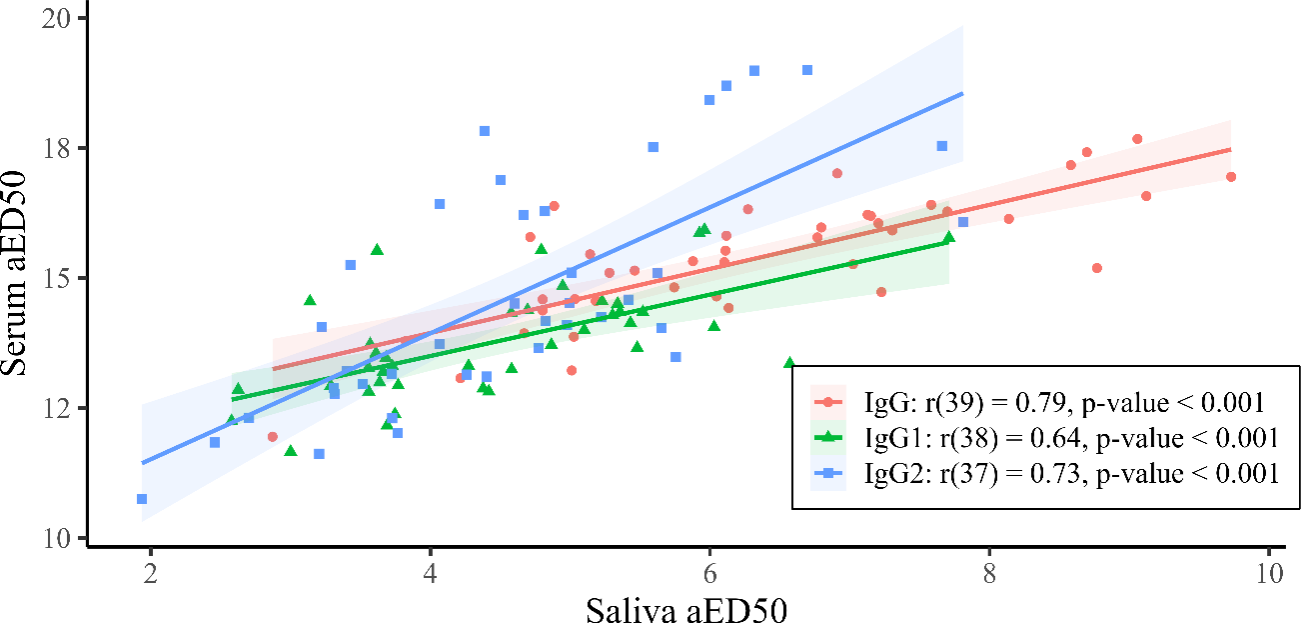
Correlation analysis of salivary and serum absolute ED50 (aED50) values for each antigen-specific response across all vaccine groups. The legend includes the Pearson’s correlation coefficient (r), the degrees of freedom in parentheses and the p-value from the correlation test for each group. Each point represents the aED50 values from saliva and serum, respectively, from a single sheep. Fitted least−squares trend lines with 95% confidence intervals (shaded regions) are shown for visual guidance only; statistical inference was based on Pearson’s correlation coefficients, which are independent of slope magnitude.

**Figure 3:**
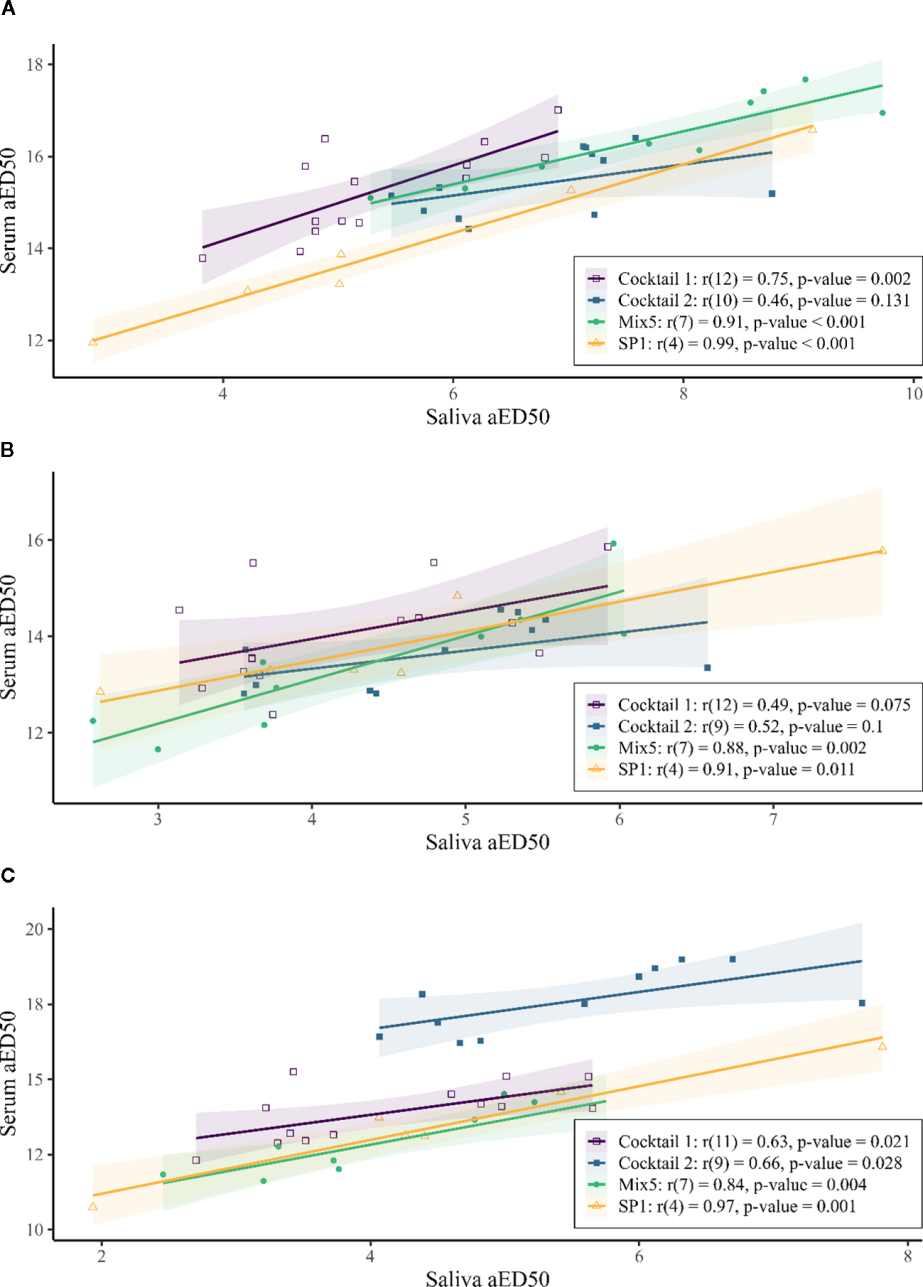
Correlation analysis of absolute ED50 (aED50) values of antigen-specific responses of IgG **(A)**, IgG1 **(B)**, and IgG2 **(C)**. Each point represents the paired serum and saliva levels from an individual sheep. The legend includes the Pearson’s correlation coefficient (r), the degrees of freedom in parentheses and the p-value from the correlation test for each vaccine antigen. The vaccine antigens used were Cocktail 1 (open squares, purple), Cocktail 2 (filled squares, blue), SP1 (open triangle, orange) and Mix5 (filled circle, green). Fitted least−squares trend lines with 95% confidence intervals (shaded regions) are shown for visual guidance only; statistical inference was based on Pearson’s correlation coefficients, which are independent of slope magnitude.

Pearson’s correlation was used to evaluate the relationship between serum and salivary antibodies in all 41 paired serum and saliva samples (**Figure 2**). There was a strong and significant positive correlation between serum aED50 and saliva aED50 for IgG, IgG1 and IgG2 (**Table 1**). Then examined within each vaccine group, serum–saliva correlations remained positive, although their strength varied across antigens (**Figure 3**). Cocktail 1 also showed a positive and statistically significant correlation (r = 0.75, p = 0.002), although weaker than the single−protein, SP1 (r = 0.99, p < 0.001), and whole−cell vaccines, Mix5 (r = 0.91, p < 0.001) (**Figure 3A**). For Cocktail 2, the correlation remained positive (r = 0.46) but did not reach statistical significance (p = 0.131), consistent with the smaller dynamic range of responses in this group. For IgG1 and IgG2, serum–saliva correlations also remained positive across vaccine groups, with variability in magnitude consistent with the underlying response ranges (**Figures 3B, C**). For IgG1 (**Figure 3B**), two groups, Mix5 (= 0.88, p = 0.002) and SP1 (r = 0.91, p = 0.011), had very strong and significant positive correlations, while Cocktail 1 IgG1 (r = 0.49, p = 0.002) and Cocktail 2 (r = 0.52, p = 0.100) had moderate positive correlations. For IgG2 (**Figure 3C**), all groups were significant with at least strong positive correlations: Cocktail 1 (r = 0.63, p = 0.021), Cocktail 2 (r = 0.66, p = 0.028), Mix5 (r = 0.84, p = 0.004), SP1 (r = 0.97, p = 0.001). Together, these pooled and within−group analyses indicate that serum and saliva show consistent relationships in antigen−specific aED50 values. These correlations complement the pattern−level concordance described above and provide supporting evidence for using serum as a practical predictor of salivary antibody responses.

### 3.3 Avidity of salivary and serum antibodies were similar for animals vaccinated with recombinant proteins

**Figure 4:**
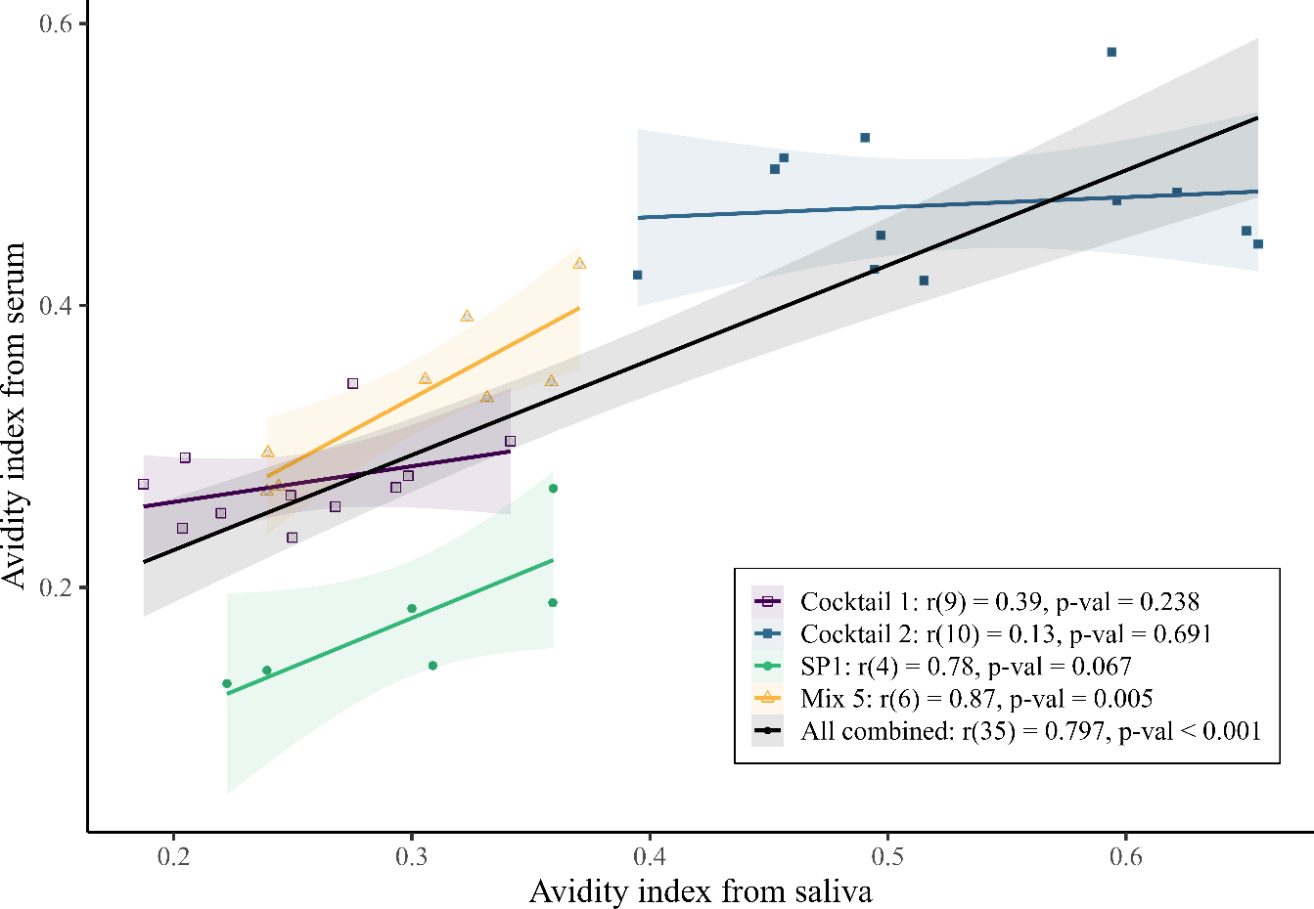
Correlations of avidity indices. The vaccine antigens were Cocktail 1 (open squares, purple), Cocktail 2 (filled squares, blue), and SP1 (open triangle, orange) and Mix5 (filled circle, green). Each point represents the paired serum and saliva avidity measured for each individual sheep. The legend includes the Pearson’s correlation coefficient (r), the degrees of freedom in parentheses and the p-value from the correlation test for each vaccine antigen, as well as for all the data combined. Fitted least−squares trend lines with 95% confidence intervals (shaded regions) are shown for visual guidance only; statistical inference was based on Pearson’s correlation coefficients, which are independent of slope magnitude.

When data from all vaccine groups were pooled, serum and salivary avidity indices showed a positive overall relationship (**Figure 4**; r = 0.80, p < 0.001). Because avidity measurements are sensitive to assay variation and constrained dynamic range—particularly for saliva—this pooled pattern was interpreted cautiously and used as supportive context rather than primary evidence. When evaluated within individual vaccine groups, correlations were variable and antigen−dependent. Mix5 showed a positive and statistically significant association between serum and salivary AI values (r = 0.87, p = 0.005), while SP1 showed a positive but non−significant trend (r = 0.74, p = 0.067). For Cocktail 1 (r = 0.39, p = 0.238) and Cocktail 2 (r = 0.13, p = 0.691), correlations remained positive in direction but were small in magnitude and not statistically significant. Avidity was not examined for IgG1 or IgG2 responses. Overall, the heterogeneity in avidity relationships highlights both the complexity of these measurements and the opportunity for future work to refine and validate avidity assessment across antigen types.

## 4 Discussion

This study was conducted to determine if serum is a good substitute for saliva to study immune and functional properties of salivary IgG, IgG1, and IgG2 in ruminants. Our findings suggest that serum can be a reliable surrogate for saliva in immunological studies. Analysis of antigen-specific IgG and its subgroups in matched serum and saliva samples from vaccinated animals revealed that higher antibody titers in serum consistently corresponded to higher titers in saliva, despite overall antibody levels being greater in serum. In addition, correlation in aED50 values between serum and salivary antibodies and analysis of paired saliva and serum samples by western blotting demonstrated similar patterns of immuno-reactivity of antibodies to antigens. While these correlations demonstrate strong covariation between serum and salivary responses, they do not imply quantitative equivalence of absolute antibody levels between these fluids. Our correlations in antibody titer and similarities in antigen specificity are consistent with studies that show the majority of IgG in saliva is derived from serum (5, 8, 9, 15, 16). Unique for our study, we also examined IgG1 and IgG2 responses, and the same correlation was observed for serum and saliva IgG1 and IgG2 antibodies, which suggest serum is consistently good for examining IgG subgroup responses in saliva.

Although IgA and IgM play important roles in mucosal immunity, particularly in immune exclusion and host–microbiome interactions, vaccine strategies designed to elicit functional antibody activity at mucosal effector sites have frequently relied on the induction of antigen-specific IgG. In ruminants, salivary IgG is derived predominantly from the systemic circulation. Parenteral vaccination therefore preferentially induces robust antigen−specific IgG responses in serum, which are continuously delivered to mucosal sites, including the oral cavity and, in ruminants, the rumen via salivary flow. Across vaccination studies targeting rumen bacteria, archaea, protozoa, and enzymes, salivary IgG has been consistently associated with binding-mediated functional outcomes predicted to enable downstream functional effects *in vivo*, such as microbial binding and inhibition of metabolic activity. In contrast, antigen−specific IgA responses are more variable and strongly influenced by mucosal vaccination route and adjuvant formulation (6, 7, 20, 24, 31). More broadly, recent reviews identify salivary IgG as the principal antibody readout linking systemic vaccination to functional activity at mucosal sites, whereas studies of salivary IgA have focused primarily on immune regulation and microbial community structure rather than vaccine−mediated inhibition (32, 33). In this context, our focus on IgG and its subclasses provides a biologically appropriate framework for evaluating the relevance of serum as a surrogate for vaccination-induced salivary antibodies.

From a practical perspective, the lower abundance of IgG in saliva does not diminish its relevance. Salivary IgG can be reliably detected and reflects systemic antigen−specific IgG responses, providing biologically meaningful information at the site of vaccine action, even where IgA predominates quantitatively (18). However, because saliva sampling is technically challenging and less amenable to high−throughput analysis, serum remains the practical matrix for large−scale screening. Demonstrating that serum IgG reliably reflects the antigen−specific IgG population present in saliva therefore supports its use as a surrogate for assessing vaccination−induced salivary antibody responses relevant to vaccine optimisation.

Pearson’s correlation coefficient was used to assess the consistency with which paired serum and salivary responses covary across animals, rather than modelling one compartment as a quantitative predictor of the other. Since correlation is independent of scale, differences in the apparent steepness of the trend lines reflect relative measurement ranges only. Accordingly, the trend lines shown in the correlation figures in this study are provided for visual guidance and were not used for statistical inference, and differences in slope values should not be interpreted as differences in correlation strength or biological linkage.

From a biological perspective, selective transport of IgG1 into the ruminant mammary gland is well established, resulting in IgG1 enriched colostrum and milk and consistent with FcRn mediated mechanisms (34–36). However, there is no evidence that FcRn preferentially transports IgG1 over IgG2 into saliva. Rather, in both sheep and humans, salivary IgG largely reflects the serum IgG subclass distribution, consistent with passive transudation from plasma (3, 37). In sheep, where IgG2 predominates in serum, salivary IgG is derived from plasma rather than local synthesis, resulting in subclass profiles that mirror those in serum (9, 36, 38). These differences we observed therefore reflect variation in the proportional transfer of IgG subclasses into saliva across the measured range, rather than evidence of selective enrichment of a particular subclass of antibody.

Our observations align with those of Hetteger et al. (16), who reported strong correlations in IgG titers between paired serum and saliva samples. A limitation of our study is that serum and saliva samples were collected at a single time point, two to three weeks after the second vaccination. Hetteger et al. (16) collected multiple samples from 20 human subjects over a six-week course and demonstrated that serum and saliva correlations persisted over this time. A strength of our study was the evaluation of more complex vaccines, comprised of a mixture of microbial cells or mixtures of recombinant proteins, in addition to testing a single protein antigen that contains a more limited repertoire of immunological epitopes. These complex antigens would be expected to elicit responses to a broader set of epitopes, more closely reflecting the diversity of targets encountered in natural infections. Analysis of the full dataset showed a positive overall relationship between serum and salivary titers across antigens, supporting the use of serum IgG as a practical indicator of salivary IgG over a wide range of responses.

Although serum can serve as a practical surrogate for saliva for certain immunological measurements, the total amount of IgG present in saliva is substantially lower than in serum. Subharat et al. (7) showed that it was possible to purify enough total IgG and antigen-specific IgG for analysis by ELISA from large volumes, e.g., 30–50 mL of saliva. In that study, the mean total serum IgG concentration in sheep (n = 30) was 19,931 µg/mL compared to a mean total salivary IgG concentration of only 41.7 μg/mL, a 478-fold difference. The relatively low concentration of IgG present in saliva is a challenge for measuring IgG antibody titers in saliva and is confounded by factors such as viscosity, differences in relative water, protein, or ion content as well as differences in salivation volume and oral health status (3, 15). In addition, background material from rumen digesta can make saliva samples from ruminants more challenging to work with than serum. Despite these challenges, robust ELISA titration curves were obtained for saliva samples, allowing aED50 values to be calculated. There was on average 500-fold difference in antigen specific responses between each serum IgG and saliva IgG pairing. This magnitude of difference is consistent with previous reports showing substantially higher IgG concentrations in serum than in saliva (5, 7, 15, 16). We focused on antigen-specific IgG and its subgroups in sheep serum and saliva, and we did not delve deeply into IgA responses in saliva. However, we did observe only minimal antigen-specific IgA responses (data not shown). Subharat et al. (7) reported that antigen-specific IgA levels stimulated by a systemic route of vaccination with a recombinant protein were lower than antigen-specific IgG levels in serum, while total IgA was close to 7-fold higher than the IgG levels observed in saliva.

In addition to antibody titer, antibody avidity is another factor that can contribute to effective immune responses. When serum and salivary avidity indices were pooled across all vaccine groups, a positive overall association was observed. However, when assessed within individual vaccine groups, avidity relationships varied substantially, with a statistically significant correlation observed only for the Mix5 group, despite a positive overall trend when data were pooled across all groups. The single recombinant protein, SP1, showed a positive but non−significant trend. This may reflect the limited sample size for this group, which reduces the power to detect moderate associations. The AI was determined using two ELISA measurements—one with and one without chaotropic agent—and it is likely that calculating avidity as a ratio of two ED50 values introduces additional noise. Given this variability, avidity comparisons between serum and saliva should be interpreted cautiously, as the strength and reliability of these relationships appear to depend on antigen type. Variability in saliva composition, including the presence of feed material, and the higher sample concentrations required for salivary IgG measurements may also contribute to this reduced consistency across vaccine groups. These factors may be overcome by using paired serum and saliva samples from a greater number of animals or by applying more robust methods for determining avidity.

To place these findings in context, it is important to consider the biological and analytical characteristics of saliva as a sample matrix. Although higher IgG avidity was observed in saliva compared with serum, this finding should be interpreted cautiously. IgG in saliva is predominantly derived from serum but is present at substantially lower concentrations and within a more dilute and compositionally altered environment than blood (3). As a result, salivary samples must be analyzed at lower dilution, conditions under which ED50−based avidity estimates are more sensitive to concentration−dependent and matrix−related assay effects. Factors such as variable fluid flow, protein loss, sampling conditions, and the heterogeneous composition of saliva can further influence antibody behavior in oral fluids. Consistent with this, antibody measurements in saliva are widely regarded as providing qualitative or comparative information rather than direct quantitative equivalents of serum measurements (3, 39). Together, these features of saliva as a sample matrix provide a plausible assay−based explanation for the higher apparent IgG avidity observed here, without implying any biologically meaningful difference in intrinsic IgG affinity between saliva and serum. Avidity measurements in this study were treated as exploratory, and conclusions regarding serum–saliva correspondence were based primarily on antibody titer, specificity, and subclass−resolved binding profiles. Functional neutralization assays were beyond the scope of this work due to saliva volume constraints, antigen complexity, and the absence of standardized assays for rumen methanogens.

Our findings indicate that antigen−specific IgG responses to single recombinant proteins, methanogen mixtures, and, as shown by Hetteger et al. (16), defined peptide epitopes, can be reliably assessed using serum samples when saliva is difficult to obtain in sufficient volume. This is especially relevant in ruminants, where collecting small volumes of serum is far more practical than obtaining the larger volumes of saliva required for many assays, particularly in studies involving large animal cohorts. A limitation of our study is that ELISA conditions were not independently optimized for each antigen. This may affect absolute signal magnitudes between antigens, although it is unlikely to influence the within−antigen serum–saliva comparisons central to our titer analyses. Avidity measurements, however, may be more sensitive to these differences. We adopted a single standardized protocol across antigens to ensure consistent within−antigen comparisons of paired serum and saliva responses. Further work will be needed to evaluate serum–saliva avidity relationships more robustly, including studies with larger sample sizes, varied vaccination schedules, alternative adjuvants, or additional methods for assessing avidity across more complex antigens.

Taken together, while saliva remains the biologically relevant compartment, serum measurements provide a reliable basis for predicting key features of antigen−specific salivary IgG responses in the context of vaccine optimization. Our study provides evidence that serum IgG can be used to investigate key immunological characteristics of salivary IgG in ruminants. This has important implications for both research and practical applications in veterinary science, particularly in situations where saliva collection is limited. Given the markedly lower levels of total and antigen−specific IgG in saliva, serum offers a practical and reliable alternative for many immunological assays. While our avidity measurements indicate that additional investigation is required before conclusions about serum–saliva avidity relationships can be made with confidence, the overall similarity in antibody–antigen binding profiles, especially for IgG, IgG1, and IgG2, supports the use of serum as a surrogate for several key salivary IgG readouts. Together, these findings enhance the flexibility and accessibility of immunological testing in ruminants, paving the way for more efficient and scalable approaches to vaccine studies and animal health monitoring.

## Data Availability

The original contributions presented in the study are included in the article/**Supplementary Material**. Further inquiries can be directed to the corresponding author.

## Supplementary material

Supplementary Figure 1 Representative SDS−PAGE analysis of vaccine antigens used in this study.

Supplementary Figure 2 Comparison of antigen-specific serum and salivary IgG.

Supplementary Figure 3 IgG1.

Supplementary Figure 4 IgG2 in sheep.

Supplementary Table 1 Details of animal trials performed in this study.

Supplementary Table 2 Tables listing the aED50 (titer log_2_) of serum and salivary for IgG.

Supplementary Table 3 IgG1.

Supplementary Table 4 IgG2.
