# Supplementary material for: Serum as a surrogate for salivary IgG: comparison of antigen-specific serum and salivary IgG in sheep
Source: Front Immunol. 2026 May 20;17:1797719. doi: 10.3389/fimmu.2026.1797719 (PMC13229843; doi:10.3389/fimmu.2026.1797719)
Supplement: Supplementary Figure 1 — Representative SDS−PAGE analysis of vaccine antigens used in this study. [file Supplementaryfile1.docx]

Supplementary Material

# Supplementary Figures and Tables

## Figures

| A |  | B |  |
| --- | --- | --- | --- |
|  | 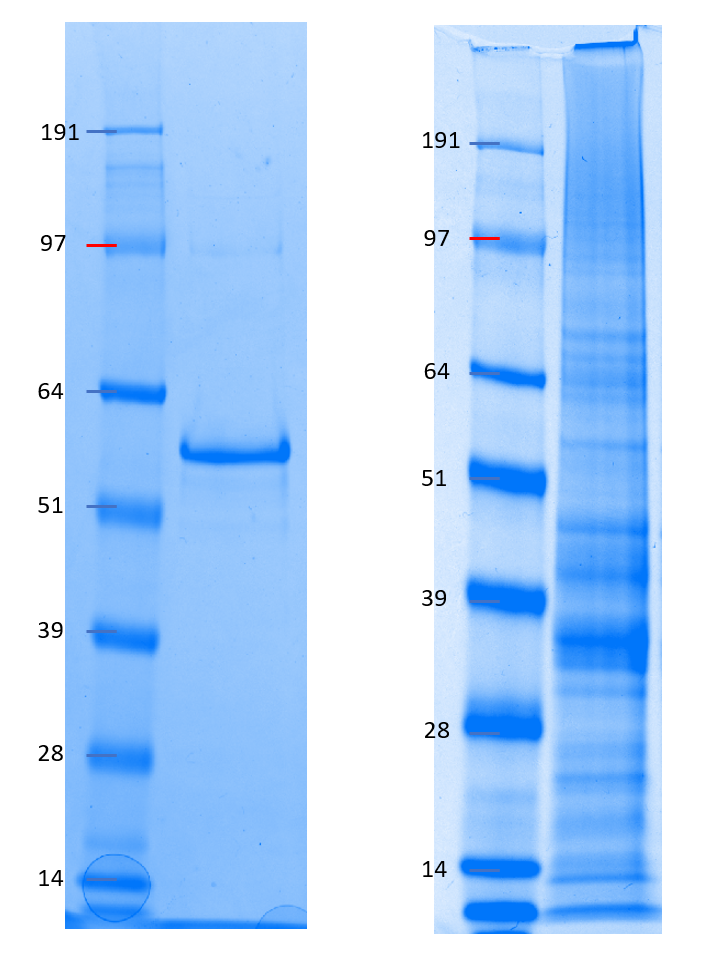 |  | 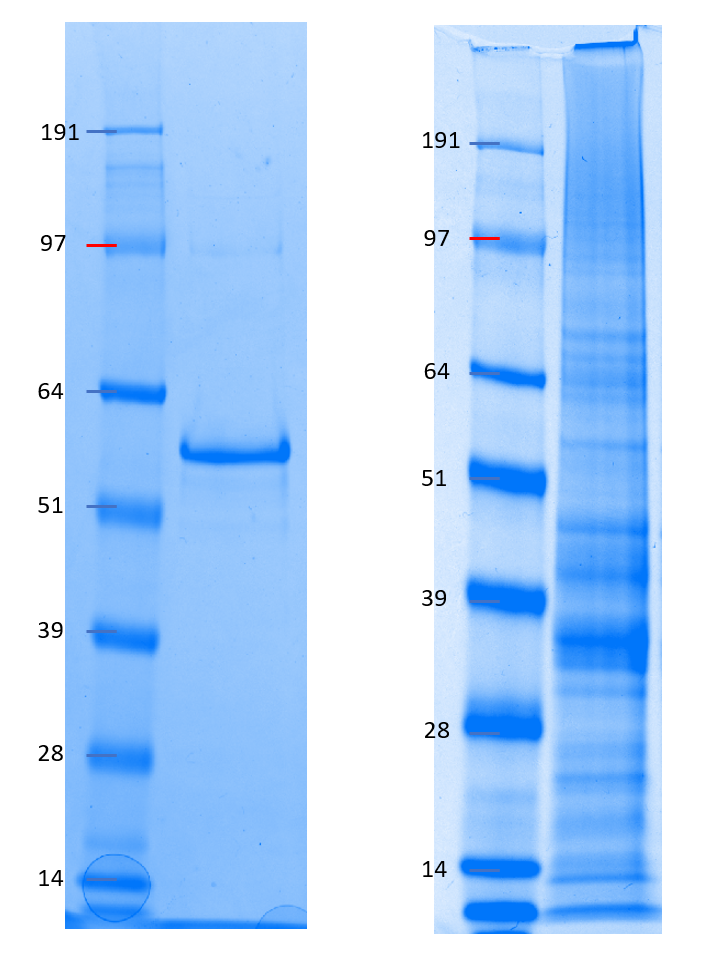 |

**Supplementary Figure 1.** Representative SDS‑PAGE analysis of vaccine antigens used in this study. (A) Recombinant SP1 protein. (B) Total cell lysate of *Methanobrevibacter ruminantium* M1. Samples (0.5 µg SP1 or 6 µg total cell lysate) were prepared in NuPAGE LDS sample buffer (Thermo Fisher Scientific) containing 50 mM dithiothreitol and incubated at 37°C for 40 min prior to electrophoresis on a 4–12% Bis‑Tris gradient gel (Thermo Fisher Scientific). SeeBlue™ Plus2 prestained protein standard (Thermo Fisher Scientific) was used as the molecular weight marker. Electrophoresis was performed in NuPAGE SDS‑MOPS running buffer at 160 V. Recombinant SP1 migrated at the expected molecular weight and showed high purity, supporting its suitability for western blotting and ELISA analyses.

| **A** | **Cocktail 1 - IgG** |  |
| --- | --- | --- |
| **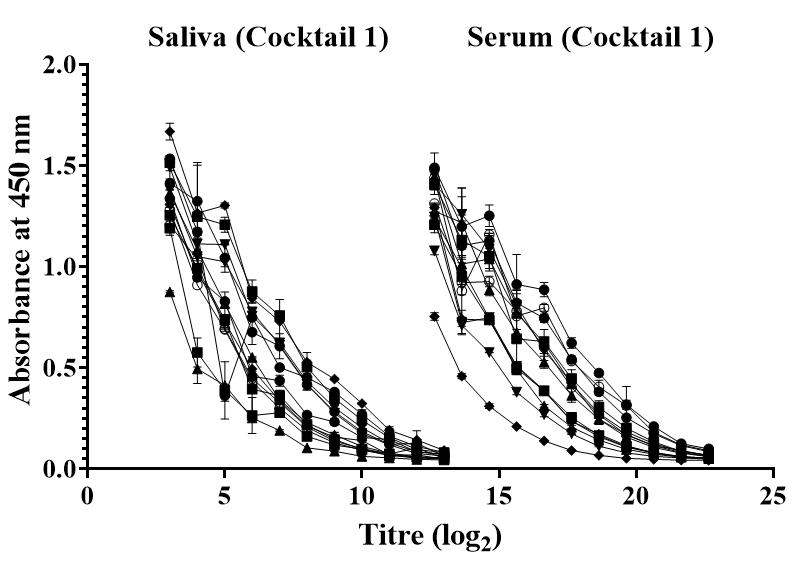** | | **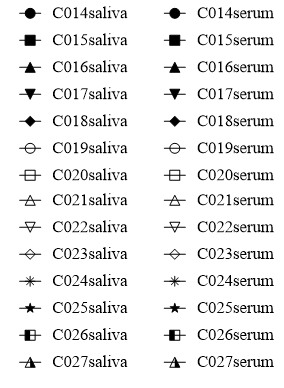** |
|  | |  |

| **B** | **Cocktail 2 - IgG** |  |
| --- | --- | --- |
| **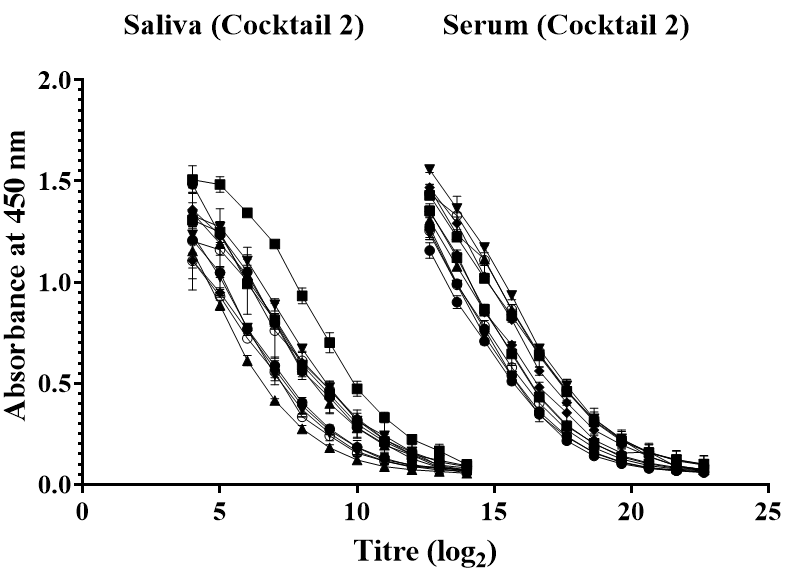** | | **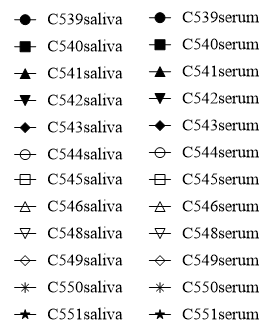** |

**Supplementary Figure 2**. ELISA titration curves of salivary IgG and serum IgG responses to Cocktail 1 (A) and Cocktail 2 (B). Means (± SE) of duplicate determinations are presented. The same symbol is used for individual animals in the titration curves with saliva and serum within each panel.

| **A** | **Cocktail 1 – IgG1** |  |
| --- | --- | --- |
| **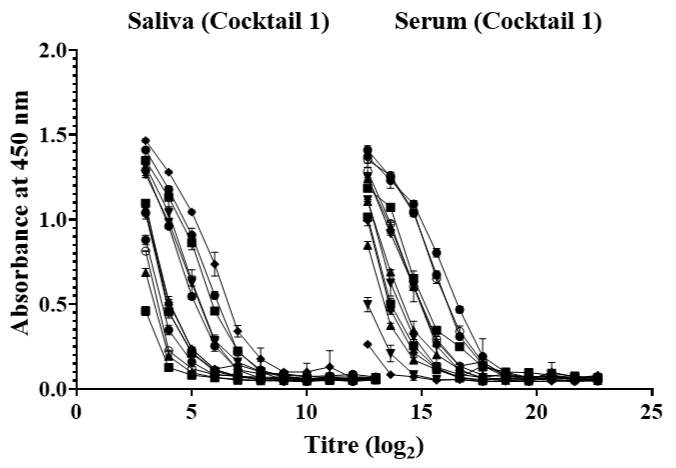** | | **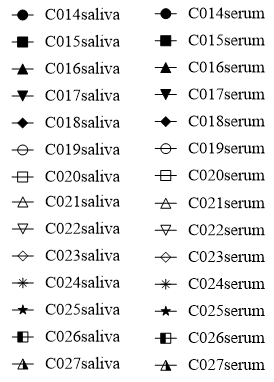** |
| **B** | **Cocktail 2 – IgG1** |  |
| **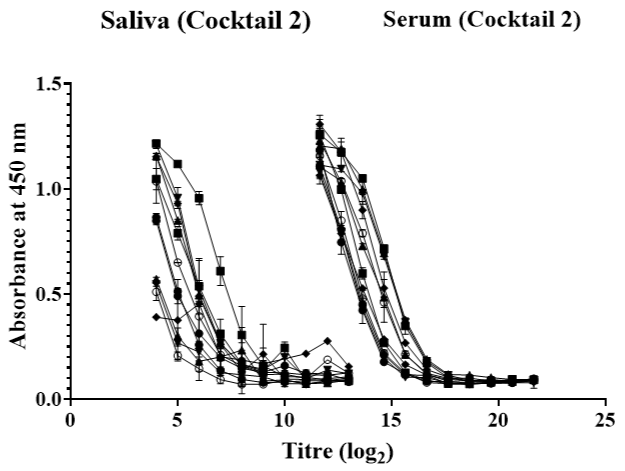** | | **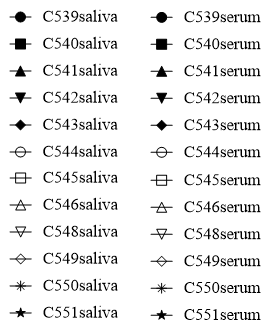** |
|  | |  |

| **C** | **SP1 – IgG1** |  |
| --- | --- | --- |
| **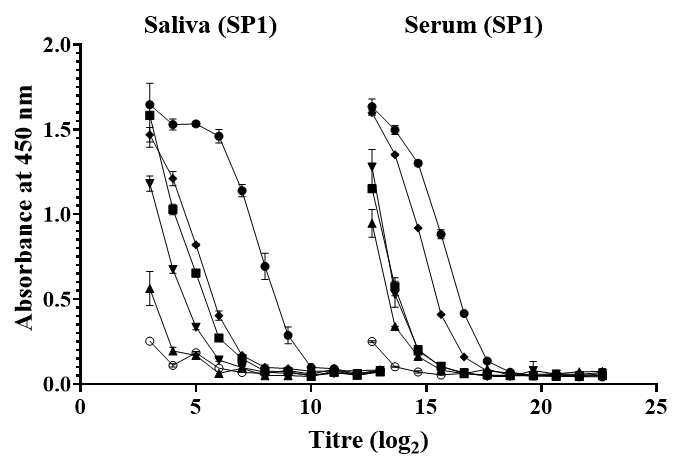** | | **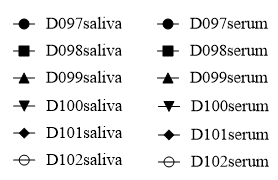** |
| **D** | **Mix5 – IgG1** |  |
| **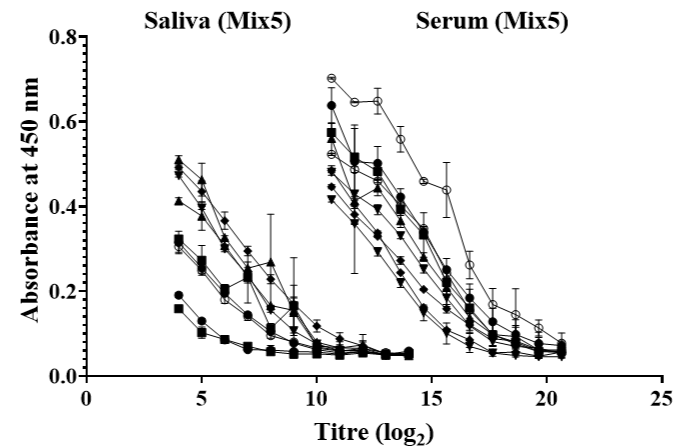** | | **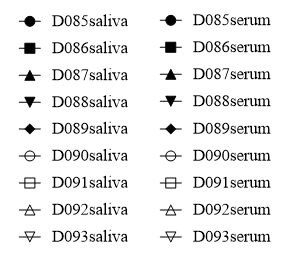** |

**Supplementary Figure 3**. IgG1 ELISA titration curves. Means (± SE) of duplicate determinations are presented. The same symbol is used for individual animals in the titration curves with saliva and serum within each panel.

| **A** | **Cocktail 1 – IgG2** |  |
| --- | --- | --- |
| **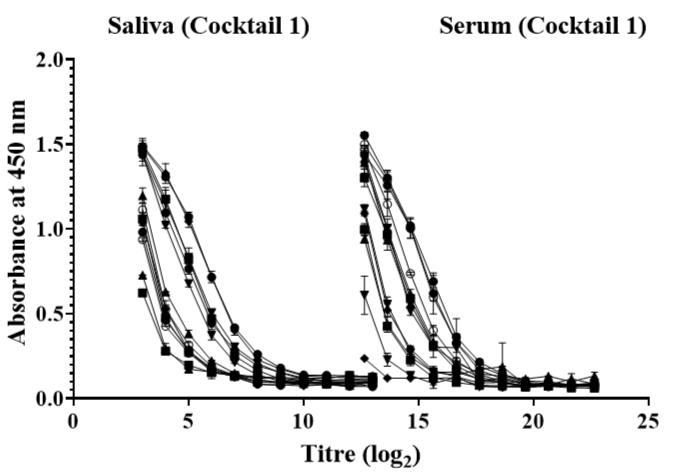** | | **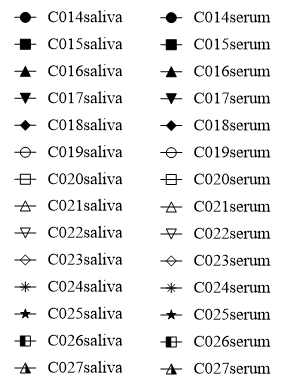** |
| **B** | **Cocktail 2 – IgG2** |  |
| **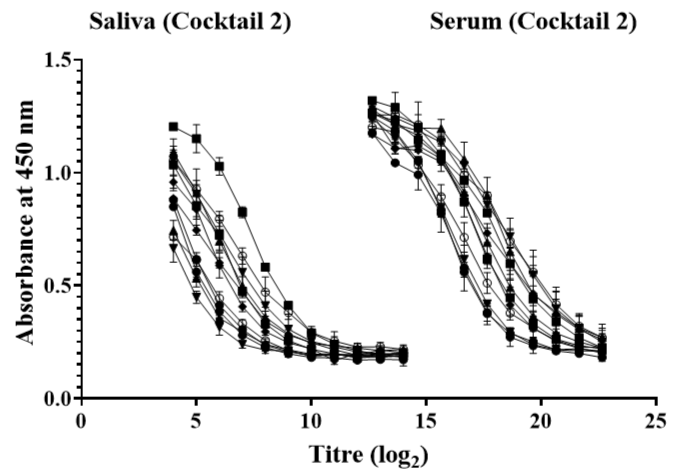** | | **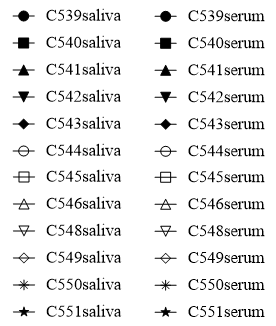** |
|  | |  |

| **C** | **SP1 – IgG2** |  |
| --- | --- | --- |
| **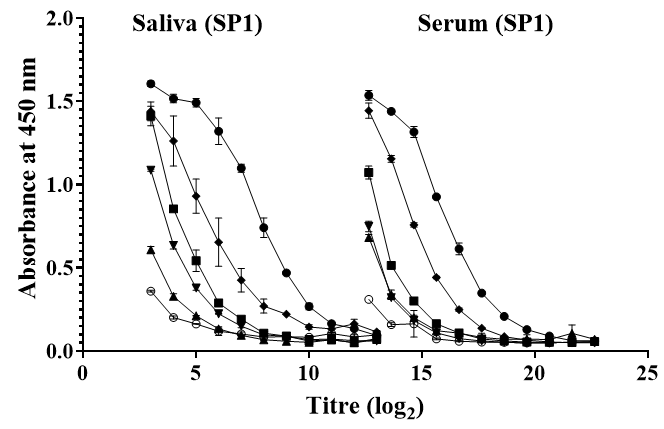** | | **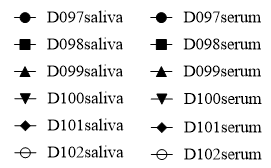** |
| **D** | **Mix5 – IgG2** |  |
| **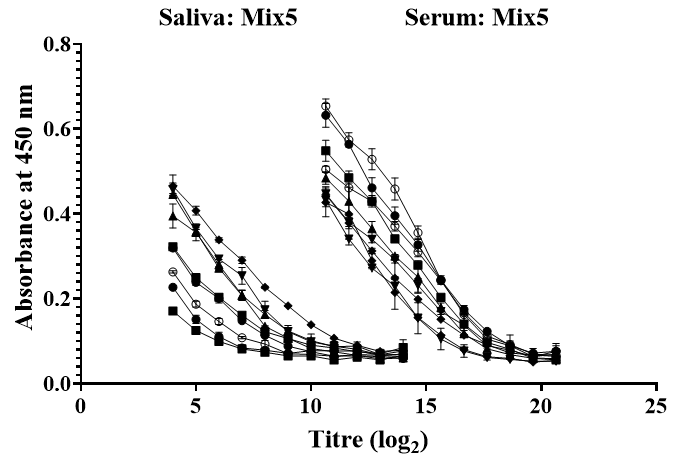** | | **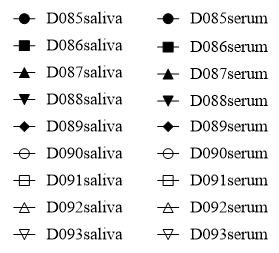** |

**Supplementary Figure 4**. IgG2 ELISA titration curves. Means (± SE) of duplicate determinations are presented. The same symbol is used for individual animals in the titration curves with saliva and serum within each panel**.**

## Supplementary Tables

**Supplementary Table 1.** Details of animal trials performed in this study

| Trial number | Vaccines used | Number of animals | Vaccination schedule | | | Animal Ethics approval |
| --- | --- | --- | --- | --- | --- | --- |
|  |  |  | Collection of serum and saliva, then vaccination 1 | Vaccination 2 | Collection of serum and saliva |  |
| 2018-3 | Cocktail 1 | 14 | 21 July 2018 | 28 August 2018 | 20 September 2018 | 14462 |
| 2019-3 | Cocktail 2 | 12 | 7 May 2019 | 5 June 2019 | 28 June 2019 | 14766 |
| 2020-1 | SP1 | 6 | 9 June 2020 | 7 July 2020 | 21 July 2020 | 14943 |
|  | Mix5 | 9 |  |  |  |  |
|  | Adjuvant only | 3 |  |  |  |  |

**Supplementary Table 2.** aED50 (titre log_2_) of serum IgG and salivary IgG

| **Group** | **Animal ID** | **aED50 serum**  **(log_2_)** | **aED50 saliva**  **(log_2_)** | **Difference (aED50 (log_2_) serum - aED50 (log_2_) saliva)** | **Difference (antilog (base2))** |
| --- | --- | --- | --- | --- | --- |
| Cocktail 1 | C014 | 14.6 | 5.03 | 9.57 | 760.08 |
| Cocktail 1 | C015 | 15.97 | 6.8 | 9.17 | 576.03 |
| Cocktail 1 | C016 | 13.94 | 4.67 | 9.27 | 617.37 |
| Cocktail 1 | C017 | 15.53 | 6.11 | 9.42 | 685.02 |
| Cocktail 1 | C018 | 16.39 | 4.88 | 11.51 | 2916.45 |
| Cocktail 1 | C019 | 14.59 | 4.80 | 9.79 | 885.29 |
| Cocktail 1 | C020 | 15.81 | 6.12 | 9.69 | 826.00 |
| Cocktail 1 | C021 | 14.38 | 4.80 | 9.58 | 765.36 |
| Cocktail 1 | C022 | 14.56 | 5.18 | 9.38 | 666.29 |
| Cocktail 1 | C023 | 16.32 | 6.27 | 10.05 | 1060.11 |
| Cocktail 1 | C024 | 17.01 | 6.91 | 10.1 | 1097.50 |
| Cocktail 1 | C025 | 15.79 | 4.71 | 11.08 | 2164.77 |
| Cocktail 1 | C026 | 15.46 | 5.14 | 10.32 | 1278.29 |
| Cocktail 1 | C027 | 13.79 | 3.82 | 9.97 | 1002.93 |
| IgG ratio Cocktail 1 | | | mean | 9.90 | 956.15 |
|  |  |  | max | 11.51 | 2916.45 |
|  |  |  | min | 9.17 | 576.03 |

| **Group** | **Animal ID** | **aED50 serum**  **(log_2_)** | **aED50 saliva**  **(log_2_)** | **Difference**  **(aED50 (log_2_) serum - aED50 (log_2_) saliva)** | **Difference (antilog (base2))** |
| --- | --- | --- | --- | --- | --- |
| Cocktail 2 | C539 | 14.73 | 7.23 | 7.5 | 181.02 |
| Cocktail 2 | C540 | 15.19 | 8.77 | 6.42 | 85.63 |
| Cocktail 2 | C541 | 15.14 | 5.46 | 9.68 | 820.30 |
| Cocktail 2 | C542 | 14.65 | 6.05 | 8.6 | 388.02 |
| Cocktail 2 | C543 | 15.92 | 7.30 | 8.62 | 393.44 |
| Cocktail 2 | C544 | 14.82 | 5.74 | 9.08 | 541.19 |
| Cocktail 2 | C545 | 14.43 | 6.13 | 8.3 | 315.17 |
| Cocktail 2 | C546 | 16.05 | 7.20 | 8.85 | 461.44 |
| Cocktail 2 | C548 | 16.20 | 7.15 | 9.05 | 530.06 |
| Cocktail 2 | C549 | 16.41 | 7.58 | 8.83 | 455.09 |
| Cocktail 2 | C550 | 15.32 | 5.88 | 9.44 | 694.58 |
| Cocktail 2 | C551 | 16.22 | 7.13 | 9.09 | 544.96 |
| IgG ratio Cocktail 2 | | | mean | 8.57 | 381.32 |
|  |  |  | max | 9.68 | 820.30 |
|  |  |  | min | 6.42 | 85.63 |

|  |  |  |  |  |  |
| --- | --- | --- | --- | --- | --- |
| **Group** | **Animal ID** | **aED50 serum**  **(log_2_)** | **aED50 saliva**  **(log_2_)** | **Difference**  **(aED50 (log_2_) serum - aED50 (log_2_) saliva)** | **Difference (antilog (base2))** |
| Mix5 | D085 | 15.10 | 5.28 | 9.82 | 903.89 |
| Mix5 | D086 | 16.14 | 8.14 | 8 | 256.00 |
| Mix5 | D087 | 17.67 | 9.06 | 8.61 | 390.72 |
| Mix5 | D088 | 17.42 | 8.70 | 8.72 | 421.68 |
| Mix5 | D089 | 16.95 | 9.73 | 7.22 | 149.09 |
| Mix5 | D090 | 15.78 | 6.77 | 9.01 | 515.56 |
| Mix5 | D091 | 16.28 | 7.70 | 8.58 | 382.68 |
| Mix5 | D092 | 15.30 | 6.10 | 9.2 | 588.13 |
| Mix5 | D093 | 17.17 | 8.58 | 8.59 | 385.34 |
| IgG ratio Mix5 | | | mean | 8.61 | 390.86 |
|  |  |  | max | 9.82 | 903.89 |
|  |  |  | min | 7.22 | 149.09 |
|  |  |  |  |  |  |
| **Group** | **Animal ID** | **aED50 serum**  **(log_2_)** | **aED50 saliva**  **(log_2_)** | **Difference**  **(aED50 (log_2_) serum - aED50 (log_2_) saliva)** | **Difference (antilog (base2))** |
| SP1 | D097 | 16.58 | 9.12 | 7.46 | 176.07 |
| SP1 | D098 | 13.87 | 5.03 | 8.84 | 458.25 |
| SP1 | D099 | 13.07 | 4.21 | 8.86 | 464.65 |
| SP1 | D100 | 13.22 | 5.01 | 8.21 | 296.11 |
| SP1 | D101 | 15.26 | 7.02 | 8.24 | 302.33 |
| SP1 | D102 | 11.95 | 2.87 | 9.08 | 541.19 |
| IgG ratio SP1 | | | mean | 8.43 | 344.90 |
|  |  |  | max | 9.08 | 541.19 |
|  |  |  | min | 7.46 | 176.07 |
| IgG ratio for all antigens | | | mean | 8.99 | 509.24 |
|  |  |  | max | 11.51 | 2916.45 |
|  |  |  | min | 6.42 | 85.63 |

**Supplementary Table 3.** aED50 (titre log_2_) of serum IgG1 and salivary IgG1

| **Group** | **Animal ID** | **aED50 serum**  **(log_2_)** | **aED50 saliva**  **(log_2_)** | **Difference**  **(aED50 (log_2_) serum - aED50 (log_2_) saliva)** | **Difference (antilog (base2)** |
| --- | --- | --- | --- | --- | --- |
| Cocktail 1 | C014 | 12.93 | 3.29 | 9.64 | 797.86 |
| Cocktail 1 | C015 | 14.29 | 5.30 | 8.99 | 508.46 |
| Cocktail 1 | C016 | 12.38 | 3.75 | 8.63 | 396.18 |
| Cocktail 1 | C017 | 14.39 | 4.69 | 9.70 | 831.75 |
| Cocktail 1 | C018 | 15.53 | 3.62 | 11.91 | 3848.29 |
| Cocktail 1 | C019 | 13.27 | 3.56 | 9.71 | 837.53 |
| Cocktail 1 | C020 | 14.33 | 4.58 | 9.75 | 861.08 |
| Cocktail 1 | C021 | 13.54 | 3.61 | 9.93 | 975.50 |
| Cocktail 1 | C022 | 13.19 | 3.66 | 9.53 | 739.29 |
| Cocktail 1 | C023 | 15.53 | 4.79 | 10.74 | 1710.26 |
| Cocktail 1 | C024 | 15.86 | 5.92 | 9.94 | 982.29 |
| Cocktail 1 | C025 | 14.55 | 3.14 | 11.41 | 2721.15 |
| Cocktail 1 | C026 | 13.66 | 5.48 | 8.18 | 290.02 |
| Cocktail 1 | C027 | 13.56 | 3.61 | 9.95 | 989.12 |
| IgG1 ratio Cocktail 1 | | | mean | 9.81 | 899.34 |
|  |  |  | max | 11.91 | 3848.29 |
|  |  |  | min | 8.18 | 290.02 |

|  |  |  |  |  |  |
| --- | --- | --- | --- | --- | --- |
| **Group** | **Animal ID** | **aED50 serum**  **(log_2_)** | **aED50 saliva**  **(log_2_)** | **Difference**  **(aED50 (log_2_) serum - aED50 (log_2_) saliva)** | **Difference (antilog (base2))** |
| Cocktail 2 | C539 | 12.82 | 4.42 | 8.40 | 337.79 |
| Cocktail 2 | C540 | 13.35 | 6.57 | 6.78 | 109.90 |
| Cocktail 2 | C541 | 13.73 | 3.57 | 10.16 | 1144.10 |
| Cocktail 2 | C542 | 12.81 | 3.56 | 9.25 | 608.87 |
| Cocktail 2 | C543 | 14.14 | 5.43 | 8.71 | 418.77 |
| Cocktail 2 | C544 | 12.99 | 3.64 | 9.35 | 652.58 |
| Cocktail 2 | C545 | 12.88 | 4.38 | 8.50 | 362.04 |
| Cocktail 2 | C546 | 14.55 | 5.23 | 9.32 | 639.15 |
| Cocktail 2 | C548 | 14.51 | 5.34 | 9.17 | 576.03 |
| Cocktail 2 | C549 | 14.34 | 5.52 | 8.82 | 451.94 |
| Cocktail 2 | C551 | 13.71 | 4.86 | 8.85 | 461.44 |
| IgG1 ratio Cocktail 2 | | | mean | 8.81 | 447.82 |
|  |  |  | max | 10.16 | 1144.10 |
|  |  |  | min | 6.78 | 109.90 |

|  |  |  |  |  |  |
| --- | --- | --- | --- | --- | --- |
| **Group** | **Animal ID** | **aED50 serum**  **(log_2_)** | **aED50 saliva**  **(log_2_)** | **Difference**  **(aED50 (log_2_) serum - aED50 (log_2_) saliva)** | **Difference (antilog (base2))** |
| Mix5 | D085 | 11.66 | 3.00 | 8.66 | 404.50 |
| Mix5 | D086 | 12.16 | 3.69 | 8.47 | 354.59 |
| Mix5 | D087 | 15.93 | 5.96 | 9.97 | 1002.93 |
| Mix5 | D088 | 14.35 | 5.36 | 8.99 | 508.46 |
| Mix5 | D089 | 14.06 | 6.03 | 8.03 | 261.38 |
| Mix5 | D090 | 13.47 | 3.68 | 9.79 | 885.29 |
| Mix5 | D091 | 12.94 | 3.77 | 9.17 | 576.03 |
| Mix5 | D092 | 12.25 | 2.58 | 9.67 | 814.63 |
| Mix5 | D093 | 14.00 | 5.10 | 8.90 | 477.71 |
| IgG1 ratio Mix5 | | | mean | 9.05 | 530.65 |
|  |  |  | max | 9.97 | 1002.93 |
|  |  |  | min | 8.03 | 261.38 |
|  |  |  |  |  |  |
| **Group** | **Animal ID** | **aED50 serum**  **(log_2_)** | **aED50 saliva**  **(log_2_)** | **Difference**  **(aED50 (log_2_) serum - aED50 (log_2_) saliva)** | **Difference (antilog (base2))** |
| SP1 | D097 | 15.77 | 7.71 | 8.06 | 266.87 |
| SP1 | D098 | 13.24 | 4.58 | 8.66 | 404.50 |
| SP1 | D099 | 12.85 | 2.62 | 10.23 | 1200.98 |
| SP1 | D100 | 13.30 | 3.73 | 9.57 | 760.08 |
| SP1 | D101 | 14.84 | 4.95 | 9.89 | 948.83 |
| SP1 | D102 | 13.31 | 4.27 | 9.04 | 526.39 |
| IgG1 ratio SP1 | | | mean | 9.21 | 592.87 |
|  |  |  | max | 10.23 | 1200.98 |
|  |  |  | min | 8.06 | 266.87 |
| IgG1 ratio for all antigens | | | mean | 9.27 | 615.41 |
|  |  |  | max | 11.91 | 3848.29 |
|  |  |  | min | 6.78 | 109.90 |

**Supplementary Table 4.** aED50 (titre log_2_) of serum IgG2 and salivary IgG2

| **Group** | **Animal ID** | **aED50 serum**  **(log_2_)** | **aED50 saliva**  **(log_2_)** | **Difference**  **(aED50 (log_2_) serum - aED50 (log_2_) saliva)** | **Difference (antilog (base2))** |
| --- | --- | --- | --- | --- | --- |
| Cocktail 1 | C014 | 12.89 | 3.31 | 9.58 | 765.36 |
| Cocktail 1 | C015 | 14.1 | 4.98 | 9.12 | 556.41 |
| Cocktail 1 | C017 | 14.51 | 4.6 | 9.91 | 962.07 |
| Cocktail 1 | C018 | 15.25 | 3.43 | 11.82 | 3615.55 |
| Cocktail 1 | C019 | 12.97 | 3.52 | 9.45 | 699.41 |
| Cocktail 1 | C020 | 14.18 | 4.82 | 9.36 | 657.11 |
| Cocktail 1 | C021 | 13.21 | 3.40 | 9.81 | 897.64 |
| Cocktail 1 | C022 | 13.16 | 3.72 | 9.44 | 694.58 |
| Cocktail 1 | C023 | 15.1 | 5.01 | 10.09 | 1089.92 |
| Cocktail 1 | C024 | 15.09 | 5.62 | 9.47 | 709.18 |
| Cocktail 1 | C025 | 14.06 | 3.22 | 10.84 | 1833.01 |
| Cocktail 1 | C026 | 14.04 | 5.65 | 8.39 | 335.46 |
| Cocktail 1 | C027 | 12.31 | 2.70 | 9.61 | 781.44 |
| IgG2 ratio Cocktail 1 | | | mean | 9.73 | 848.91 |
|  |  |  | max | 11.82 | 3615.55 |
|  |  |  | min | 8.39 | 335.46 |

|  |  |  |  |  |  |
| --- | --- | --- | --- | --- | --- |
| **Group** | **Animal ID** | **aED50 serum**  **(log_2_)** | **aED50 saliva**  **(log_2_)** | **Difference**  **(aED50 (log_2_) serum - aED50 (log_2_) saliva)** | **Difference (antilog (base2))** |
| Cocktail 2 | C539 | 16.28 | 4.82 | 11.46 | 2817.11 |
| Cocktail 2 | C540 | 17.54 | 7.66 | 9.88 | 942.27 |
| Cocktail 2 | C541 | 17.83 | 4.39 | 13.44 | 11113.30 |
| Cocktail 2 | C542 | 16.42 | 4.07 | 12.35 | 5220.60 |
| Cocktail 2 | C543 | 17.51 | 5.59 | 11.92 | 3875.05 |
| Cocktail 2 | C544 | 16.89 | 4.5 | 12.39 | 5367.37 |
| Cocktail 2 | C545 | 16.22 | 4.67 | 11.55 | 2998.45 |
| Cocktail 2 | C546 | 18.42 | 6.00 | 12.42 | 5480.15 |
| Cocktail 2 | C548 | 18.7 | 6.12 | 12.58 | 6122.90 |
| Cocktail 2 | C549 | 18.99 | 6.32 | 12.67 | 6517.03 |
| Cocktail 2 | C551 | 19 | 6.70 | 12.30 | 5042.77 |
| IgG2 ratio Cocktail 2 | | | mean | 12.05 | 4252.06 |
|  |  |  | max | 13.44 | 11113.30 |
|  |  |  | min | 9.88 | 942.27 |

|  |  |  |  |  |  |
| --- | --- | --- | --- | --- | --- |
| **Group** | **Animal ID** | **aED50 serum**  **(log_2_)** | **aED50 saliva**  **(log_2_)** | **Difference**  **(aED50 (log_2_) serum - aED50 (log_2_) saliva)** | **Difference (antilog (base2))** |
| Mix5 | D085 | 11.62 | 3.20 | 8.42 | 342.51 |
| Mix5 | D086 | 12.02 | 3.76 | 8.26 | 306.55 |
| Mix5 | D087 | 14.52 | 5.00 | 9.52 | 734.19 |
| Mix5 | D088 | 14.25 | 5.22 | 9.03 | 522.76 |
| Mix5 | D089 | 13.49 | 5.76 | 7.73 | 212.31 |
| Mix5 | D090 | 12.77 | 3.31 | 9.46 | 704.28 |
| Mix5 | D091 | 12.3 | 3.73 | 8.57 | 380.04 |
| Mix5 | D092 | 11.84 | 2.46 | 9.38 | 666.29 |
| Mix5 | D093 | 13.66 | 4.77 | 8.89 | 474.41 |
| IgG2 ratio Mix5 | | | mean | 8.83 | 456.51 |
|  |  |  | max | 9.52 | 734.19 |
|  |  |  | min | 7.73 | 212.31 |
|  |  |  |  |  |  |
| **Group** | **Animal ID** | **aED50 serum**  **(log_2_)** | **aED50 saliva**  **(log_2_)** | **Difference**  **(aED50 (log_2_) serum - aED50 (log_2_) saliva)** | **Difference (antilog (base2))** |
| SP1 | D097 | 16.08 | 7.81 | 8.27 | 308.69 |
| SP1 | D098 | 13.14 | 4.26 | 8.88 | 471.14 |
| SP1 | D099 | 13.73 | 4.06 | 9.67 | 814.63 |
| SP1 | D100 | 13.11 | 4.40 | 8.71 | 418.77 |
| SP1 | D101 | 14.58 | 5.42 | 9.16 | 572.05 |
| SP1 | D102 | 10.75 | 1.93 | 8.82 | 451.94 |
| IgG2 ratio SP1 | | | mean | 9.04 | 526.97 |
|  |  |  | max | 9.67 | 814.63 |
|  |  |  | min | 8.71 | 418.77 |
| IgG2 ratio for all antigens | | | mean | 10.06 | 1063.85 |
|  |  |  | max | 13.44 | 11113.30 |
|  |  |  | min | 7.73 | 212.31 |
